# Psilocybin as Transformative Fast‐Acting Antidepressant: Pharmacological Properties and Molecular Mechanisms

**DOI:** 10.1111/fcp.70038

**Published:** 2025-07-16

**Authors:** Makiath Adebo, Mathilda Bonnet, Ons Laouej, Celine Defaix, Josephine C. McGowan, Florence Butlen‐Ducuing, Denis J. David, Erwan Poupon, Laurent Tritschler, Alain M. Gardier

**Affiliations:** ^1^ Université Paris‐Saclay, Inserm CESP/UMR 1018, MOODS Team Orsay France; ^2^ Department of Psychiatry Harvard Medical School, McLean Hospital Belmont Massachusetts USA; ^3^ Institut Gustave Roussy, Psycho‐Oncology Unit Interdisciplinary Department for the Organization of Patient Pathways, Cancer Campus Grand Paris France; ^4^ Université Paris‐Saclay, UVSQ, Inserm, CESP/UMR 1018, MOODS Team Villejuif France; ^5^ Université Paris‐Saclay, Faculté de Pharmacie, CNRS, BioCIS Orsay France

**Keywords:** antidepressant, humans, psilocin, psilocybin, psychedelics, rodents

## Abstract

In the 1950s–60s, serotonergic psychedelic drugs were studied as potential adjuvants to psychotherapy to treat addiction and alcoholism. However, starting in the 70s, preclinical and clinical studies on psychedelics stopped for decades because legislation controlled its recreational use, citing their hallucinogenic and psychotomimetic effects, as well as their abuse potential. Amazingly, we are witnessing an impressive return of these drugs due to recent clinical trials suggesting a therapeutic potential of psychedelics, among them psilocybin, for treating patients with depression resistant to conventional antidepressant drugs. Yet, their underlying mechanisms of action remain incompletely elucidated. This review provides an update on seminal clinical trials using psilocybin, as well as preclinical work uncovering the pharmacological properties and experimental pharmacology of psilocybin and its active metabolite psilocin. These drugs are primarily serotonin 5‐HT_2A_ receptor (5‐HT2AR) agonists. Although there is a consensus that 5‐HT2AR activation mediates its psychedelic effects in human and rodent models of anxiety/depression, its role in psilocin's antidepressant effects remains controversial. This review also provides an overview of neurotransmitter systems, neuroplasticity, and neural circuits activated by psilocin. Further research in developing effective antidepressants for depression is prescient now more than ever, as according to the World Health Organization (WHO), depression will be the main cause of disability in 2030. Understanding the mechanisms through which psilocybin/psilocin would be an effective antidepressant is crucial to ultimately validate its therapeutic potential when combined with SSRIs/SNRIs in mood disorders.

**Abbreviations**
Ach: acetylcholine
AMPA: α‐amino‐3‐hydroxy‐5‐methyl‐4‐isoxazolepropionic acid
AMPA‐R: α‐amino‐3‐hydroxy‐5‐methyl‐4‐isoxazolepropionic acid receptor
BBB: blood–brain barrier
BDNF: brain‐derived neurotrophic factor
CMMS: chronic multimodal stress
CDM: chronic behavioral despair model
DA_ext_
: dopamine extracellular
DAG: diacylglycerol
DMT: dimethyltryptamine
DOI: 2,5‐Dimethoxy‐4‐iodo‐amphetamine
DRN: dorsal raphe nucleus
ED50: median effective dose
Egr1: early growth response protein 1
Egr2: early growth response protein 2
EMA: European Medicines Agency
EPM: elevated plus maze
FDA: Food and Drug Administration
fMRI: functional magnetic resonance imaging
FST: forced swim test
GABA_ext_
: gamma‐aminobutyric acid extracellular
GLU_ext_
: glutamate extracellular
GPCR: G protein‐coupled receptors
HEK 293 cells: immortalized human embryonic kidney cells
HI: hippocampus
HTR: head‐twitch response
i.p: intraperitoneal
i.v: intravenous
IP3: inositol 1,4,5‐trisphosphate
IκBα: B‐cells inhibitor alpha
KO: knockout
LSD: lysergic acid diethylamide
MDD: major depressive disorder
mPFC: medial prefrontal cortex
mRNA: messenger ribonucleic acid
mTOR: mammalian target of rapamycin
NMDA: *N*‐methyl‐D‐aspartate
NMDA‐R: *N*‐methyl‐D‐aspartate receptor
NSF: novelty suppressed feeding test
NTRK2: neuronal receptor tyrosine kinase‐2
OF: open field
PET: positron emission tomography
PLC: phospholipase C
PSD‐95: postsynaptic density protein 95 (PSD‐95)
PV: parvalbumin
RT‐PCR: reverse transcription polymerase chain reaction
SNP: single nucleotide polymorphisms
SNRI: serotonin norepinephrine reuptake inhibitor
SSRI: selective serotonin reuptake inhibitor
TRD: treatment‐resistant depression
TrkB: tyrosine receptor kinase B receptor
TST: tail suspension test
VTA: ventral tegmental area
WHO: World Health Organization
WT: wild type
18F‐FDG: 18F‐fluorodeoxyglucose
5‐HT: serotonin, 5‐hydroxytryptamine
5‐HT1AR: 5‐HT_1A_ serotonin receptor subtype
5‐HT2AR: 5‐HT_2A_ serotonin receptor subtype

## 1 Introduction

Psychedelics have been used for millennia by humans for a large variety of cultural purposes. In more recent times, these drugs have been synthesized in the laboratory; for instance, mescaline was first extracted from the peyote cactus by Arthur Heffter in 1897, while lysergic acid diethylamide (LSD) from ergot was synthesized by Albert Hoffman in 1943. After their rise in the 60s, hallucinogen psychedelics were then banned for decades to regulate their recreational and medical uses [1].

Recently, there has been a renewal of human research on psychedelic drugs, such as psilocybin, to treat several psychiatric diseases [2]. Recent phase II clinical trials suggested that psilocybin can produce rapid and sustained antidepressant effects following one or two administrations in patients with treatment‐resistant depression (TRD) [3, 4, 5]. This is an important issue knowing that depression is a serious public health issue; it has been named the most common psychiatric disorder worldwide, affecting nearly 350 million people. It is widely accepted that mood disorders greatly reduce quality of life [6]. First‐line treatments for depression (i.e., classical monoaminergic antidepressants such as serotonin selective reuptake inhibitors [SSRIs] and mixed serotonin noradrenaline reuptake inhibitors [SNRIs]) have a delayed onset of action of several weeks, with low response rates and with numerous adverse effects, further highlighting the need for more effective, rapid‐acting therapeutics.

There are a wealth of drugs classified as psychedelics that may be useful for the clinic, but their downstream actions diverge. The classification of psychedelics includes natural products from plants, e.g., dimethyltryptamine (DMT) composing ayahuasca, the indole alkaloid ibogaine made from the bark of the shrub *Tabernanthe iboga*, mescaline, or indolamines from mushrooms, e.g., psilocybin [7]. Other LSD‐like serotonergic psychedelics are synthetic drugs including phenethylamines or tryptamines, e.g., 2,5‐dimethoxy‐4‐iodo‐amphetamine (DOI) [8]. This list includes synthetic compounds such as ketamine, a dissociative anesthetic agent and *N*‐methyl‐d‐aspartate (NMDA) glutamate receptor antagonist, though it is important to note that ketamine (i) its chemical structure links it to the phenethylamines' family, and (ii) is formally classified as a dissociative anesthetic hallucinogen rather than as a psychedelic. To date, it is known that a single dose of ketamine induces a rapid increase in dendritic spine density (i.e., synaptogenesis) in the medial prefrontal cortex (mPFC), which may underlie its sustained antidepressant effect [9].

Serotonergic psychedelics are distinct because they produce a unique profile of subjective effects in humans, e.g., alteration of a person's thoughts, feelings, awareness of their own surroundings, perception, emotion, and cognition [10]. Drugs such as psilocin and LSD can induce these effects via activation of the 5‐HT_2A_ serotonin receptor subtype (5‐HT2AR) [11]. There is a consensus that cortical 5‐HT2AR activation mediates the hallucinogenic effects of LSD and psilocybin in humans [12] and mice [13]. However, the exact role of 5‐HT2AR in their antidepressant effects and the additional role of other serotonin receptors across species remain controversial and an active area of research (reviewed below) [14, 15].

This review will focus on one of these serotonergic psychedelics, psilocybin, a pro‐drug quickly metabolized in the jejunum and colon in its active metabolite psilocin (4‐OH‐dimethyltryptamine), a non‐selective 5‐HT2AR agonist and classic “psychedelic” drug [16]. Both compounds occur naturally in the “psilocybe”, “hallucinogenic” mushrooms and are structurally related to the endogenous neurotransmitter serotonin (5‐OH‐tryptamine, 5‐HT). Care will be taken in the review to compare recently discovered effects of psilocybin to the wealth of clinical and preclinical literature on another rapid‐acting antidepressant: ketamine. In our lab, we have extensively investigated ketamine's mechanism of action in vivo in an animal model of anxiety‐depression [17, 18]. Having this knowledge, (*R,S*)‐ketamine, a racemic mixture, will be viewed as the compound of reference. (*R,S*)‐ketamine promotes neuroplasticity, a key mechanism of its effectiveness [9, 19, 20]. In 2019, one of its enantiomers, esketamine SPRAVATO nasal spray formulation has been the first US Food and Drug Administration (FDA) and European Medicines Agency (EMA)‐approved glutamatergic fast‐acting antidepressant medication in TRD (https://www.fda.gov/) in combination with an SSRI/SNRI antidepressant drug [21]. While initial targets of psilocybin vs. ketamine differ (i.e., psilocin increases serotonergic signaling while ketamine is a glutamatergic modulator), evidence suggests that the downstream mechanisms of action of both compounds converge and may explain why ketamine and potentially psilocin exert rapid antidepressant‐like effects [10, 22].

Although serotonergic psychedelics such as psilocybin and some phenethylamines, such as ketamine, attract our attention as fast‐acting antidepressant drugs, as shown in a recent PubMed analysis showing an increasing number of preclinical publications [23], we still need to understand their precise molecular and cellular mechanisms of action in order to develop more targeted drugs for different forms of depression.

This review aims to synthesize the growing clinical and preclinical data on psilocybin and psilocin, their pharmacological properties, neurotransmitter systems, neuroplasticity, and neural circuits activated by psilocin. It is the right time to do so because in recent news from the November 5th, 2024 election, American citizens in Massachusetts (MA) voted against legalizing the cultivation, consumption, and possession of natural psychedelics: psilocybin, psilocin, dimethyltryptamine (DMT). Of the 50 states and the District of Columbia, only Oregon and Colorado legalized them, the ramifications of which remain to be seen. Understanding the mechanisms through which psilocybin/psilocin acts as an effective antidepressant is crucial to validating its therapeutic effectiveness as an alternative to SSRIs/SNRIs in mood disorders, further informing the development of alternative drugs, and gaining a better understanding of what patients may benefit the most from these treatments.

## 2 Preliminary Clinical Data

The sociopolitical environment of the 1970s resulted in halting research into psilocybin's therapeutic use for decades. The renewed interest in psilocybin in recent years marks a turning point in psychopharmacology.

One of the first dose‐effect studies described in hallucinogen‐naïve adults that psilocybin (5–30 mg/70 kg, p.o.) can occasion mystical‐type experiences that have persistent positive effects on mood [24]. Then, several imaging studies using functional magnetic resonance imaging (fMRI) were performed in the human brain, first in healthy volunteers (reviewed in more detail below, Section 3.2) [25]. Potential therapeutic effects of psilocybin were then analyzed in patients with moderate‐to‐severe unipolar TRD receiving two oral doses of psilocybin (10 and 25 mg, 7 days apart) in a supportive setting [3, 26]. Psilocybin's treatment was safe and effective in this preliminary study and motivated further clinical trials in TRD.

The clinical trials described above were carried out “open‐label”. In 2023, a team from King's College London and affiliated to COMPASS, a Biotechnology Company, published the results of a “double‐blind, randomized” phase 2 clinical trial, comparing the antidepressant effect of psilocybin to that of different SSRIs, a conventional serotonergic antidepressant drug [27]. The effectiveness of a single 25 mg dose of psilocybin alongside psychological support and a followed up for 3 weeks was evaluated as a reduction of more than 50% in the depression score. Such a reduction occurs 22% more in psilocybin‐treated patients than in SSRI‐treated ones. Although carried out with a small number of patients, these results are encouraging.

Since then, extended results have shown that psilocybin is effective in treating patients with cancer and depression [28], in major depressive disorders (MDD) [29], and in patients with TRD taking psilocybin with a concomitant SSRI medication [27] or alone [5]. Amazingly, in this latter Phase 2 trial involving participants with TRD, psilocybin at a single dose of 25 mg reduced depression scores significantly more than a 1‐mg dose over a period of 3 weeks but was associated with adverse effects [5].

Such successful clinical studies suggest psilocybin's potential to treat TRD with rapid and prolonged antidepressant effectiveness, thus leading to its designation as a breakthrough innovation (breakthrough therapy) by the FDA in 2019, an action that is meant to accelerate the process of drug development. However, regarding benefit/risk ratio of psilocybin in depressive symptoms, these studies are preliminary because the majority of them have not clearly established whether it was psilocin or the therapeutic engagement of patients that produced the reported beneficial effects [30]. In May 2024, the EMA organized a meeting for researchers and journalists “t*o revisit the therapeutic potential of psychedelic compounds for mental health conditions such as treatment‐resistant depression, addictive disorders and post‐traumatic stress* “. It highlights the emergency to develop new therapeutic strategies for depression since it will be the leading cause of disability in 2030 according to the World Health Organization (WHO, https://www.who.int/health‐topics/depression).

However, several questions remain regarding the inclusion of adequate control groups (against placebo or midazolam?) that mimic psychedelic effects without the therapeutic benefits and follow‐up measurements in larger clinical trials. As a parallel, the rise of esketamine as a nasal spray (SPRAVATO) received rapid approval from the FDA and EMA. Yet, the lack of controls and adequate sample sizes revealed that the effects of this nasal spray were moderate to nonexistent. Thus, caution should be taken in designing clinical trials for psilocybin and psilocin. Systematic reviews and meta‐analyses have also examined the benefits (response, remission rates) of psilocybin combined with psychotherapy for depression [31, 32, 33]. In sum, future clinical trials should aim to optimize psilocybin dosing (as well as test the efficacy of microdosing) and minimize the likelihood of functional unblinding by including active comparators (see https://classic.clinicaltrials.gov/), while also carefully considering the beneficial role of psychotherapy alongside treatment.

## 3 Basics of Psilocybin and Psilocin Pharmacology

### 3.1 Serotonin 5‐HT2AR

There are many drugs that are considered agonists of the 5‐HT2AR, and their potency and efficacy vary between assays. 5‐HT2AR evokes both G‐protein‐coupled and β‐arrestin2‐mediated signaling pathways. A biased agonist at this receptor could offer the potential of an antidepressant devoid of hallucinogenic effects, as it has been hypothesized that the hallucinogenic effects result from selective activation of 5‐HT2AR signaling pathways (see Section 3.1.4).

#### 3.1.1 Affinity

Classical hallucinogens belong to three main chemical classes: the tryptamines (e.g., psilocybin/psilocin, DMT), serotonin (5‐HT), phenethylamines (e.g., DOI, mescaline), and semi‐synthetic ergolines (e.g., LSD). They all display high affinity for the 5‐HT2AR, but they also bind to a lesser extent to 5‐HT1, 5‐HT4, 5‐HT5, 5‐HT6, and 5‐HT7 receptor subtypes [34], with less affinity to some non‐serotonergic receptors [10, 11]. Table 1 shows a summary of binding parameters [Ki (nM)/pKi] of some classical hallucinogens being 5‐HT2AR ligands.

**TABLE 1:** Affinity of 5‐HT2AR ligands in various cellular models.

18/03/2025			
Affinity properties			
Ligands	Ki	pKi	Model
**5‐HT**	= 1124 ± 239 nM [35] = 4.84 ± 0.2 nM [36] = 77.6 ± 13.8 nM [36] = 7.3 ± 2,69 nM for the high affinity binding site [37] = 52.50 ± 11.42 nM for the medium affinity binding site [37] = 198.13 ± 55.32 nM in [37]	= 7.79 ± 0.03 [38]	COS‐7 cells transiently transfected with rat 5‐HT2AR using Fugene6 transfection reagent [35] Human embryonic kidney (HEK) 293 with high stable expression of wild‐type5‐HT2AR [36] HEK 293 cells expressing human 5‐HT2AR [38] Brain cortex from Postmortem brain tissue “normal” male adult died in automobile accident (young and old adults) [37]
**Psilocybin**	= 2096 nM [39]	—	C57BL/6J mice brains membranes [39]
**Psilocin**	= 11.8 ± 1.2 nM [36] = 22.8 ± 4.0 [36] = 0.049 ± 0.01 μM [40] = 173 nM [41] = 120 nM [41] = 478 nM [41] = 180 nM [39] = 235 nM [39]	= 6. 741 ± 0.186 in mouse brain cortex [41] = 6.885 ± 0.051 in human brain cortex [41] = 6.256 ± 0.244 in human 5‐HT2AR expressing cells [41]	Human embryonic kidney (HEK) 293 with high stable expression of wild‐type human 5‐HT2AR [36] Human embryonic kidney (HEK) cells overexpressing human 5‐HT2AR [40] Bilateral brain cortices of adult male C57BL/6J mice (8 weeks old) [41] Human dorsolateral prefrontal cortex (on the basis of the absence of neuropsychiatric disorders) [41] CHO‐K1 cells expressing human 5‐HT2AR [41] Cells transfected with human 5‐HT2AR [39] C57BL/6 J mouse brain membranes [39]
**Norpsilocin**	= 391 nM [39] = 706 nM [39]	—	Cells transfected with human 5‐HT2AR [39] C57BL/6J mouse brain membranes [39]
**DOI**	= 78.5 ± 25.6 nM [35] = 0.58 ± 0.06 nM [36] = 3.36 ± 0.91 nM [38] = 11 nM [39] = 0.7 nM [42]	= 9.03 ± 0.11 [38] = 9.19 ± 0.12 [38]	COS‐7 cells transiently transfected with rat 5‐HT2AR using Fugene6 transfection reagent [35] NIH‐3 T3 cells stably expressing rat 5‐HT2AR [36] HEK 293 cells expressing human 5‐HT2AR [38] Cells transfected with human 5‐HT2AR [39] Rat brain homogenates [42]
**Ketanserin**	= 1.7 ± 0.29 nM [35] = 2.3 ± 0.42 nM [35] = 2.88 ± 0.97 nM [38]	= 8.09 ± 0.09 [38]	COS‐7 cells transiently transfected with rat 5‐HT2AR using Fugene6 transfection reagent [35] HEK 293 cells expressing human 5‐HT2AR [38]
**MDL 100907 = Volinanserin**	—	= 8.73 ± 0.20 [38]	HEK 293 cells expressing human 5‐HT2AR [38]
**Spiperone**	= 0.71 ± 0.09 nM to displace 1 nM [3H]‐Ketanserin [35]	= 7.81 ± 0.29 [38]	COS‐7 cells transiently transfected with rat 5‐HT2AR using Fugene6 transfection reagent [35] HEK 293 cells expressing human 5‐HT2AR [38]

*Note:* Green = 5‐HT2AR agonist.; Orange = 5‐HT2AR antagonist.

Abbreviations: HTR = head‐twitch response behaviors, ICSS = intracranial self‐stimulation, PI = phosphatidylinositides.

Previous studies in humans reported that a 50% to 70% 5‐HT2AR occupancy level was required for an intense psilocybin‐induced psychological experience [43].

Species‐specific differences in amino acids, e.g., at residue Ser242, in the binding pocket of psychedelics to the human 5‐HT2AR have been described [44, 45]. Mutating Ser242 to the rodent residue Ala242 (thereby creating a humanized receptor in the rodent) accelerates the dissociation rate of ergolines or tryptamines from 5‐HT2AR binding [11]. These studies suggest that the Ala/Ser242 species variation in amino acid sequence accounts for the structure–activity relationship of psychedelics. These species variations likely induce distinct antidepressant‐like effects of serotonergic psychedelics in behavioral tests performed in rodent models and should be carefully considered when designing rodent studies to test for psychedelic efficacy.

In postmortem tissue, 5‐HT2AR expression, protein immunoreactivity, and functionality have been studied in the mPFC [46]. Using RT‐PCR in human postmortem brain, the highest levels of 5‐HT2AR mRNA were found in cerebral cortical areas [47]. Furthermore, the existence of constitutively active 5‐HT2AR was described in the human mPFC [48]. These data reveal a potential mode of action of these drugs in the mPFC and surrounding cortical areas.

In the rat cortex, 5‐HT2AR are expressed mainly in pyramidal cells, with sparse expression in parvalbumin (PV)‐expressing interneurons [49]. In pyramidal cells, 5‐HT2AR are complexed with various scaffolding proteins, including postsynaptic density (PSD)‐95.

#### 3.1.2 Efficacy

Serotonergic psychedelics produce a unique profile of subjective effects in humans. The 5‐HT2AR in the mPFC is important for the acute hallucinogenic effects of psychedelic drugs and for atypical antipsychotic drug actions [50]. Animal behavioral models cannot precisely capture these perturbations of perception, cognition, and mood produced by psychedelics in humans [51]. To evaluate the role of the G(q) signaling pathway and of the 5‐HT2AR in general in psychedelics‐induced behaviors, a behavioral model of 5‐HT2AR activation is often used: induction of head‐twitch response (HTR) is recognized as a behavioral *proxy* in rodents of psychedelic‐induced hallucinations in humans [52]. HTR dose–response was absent when psychedelics (LSD, DOI, psilocin) were administered to 5‐HT2AR knock‐out mice [53], highlighting the critical role of this receptor for hallucinogenic properties. Lisuride, another 5HT2AR agonist, lacks comparable psychoactive properties in humans and fails to induce the HTR behavior in mice [13]. Consequently, a correlation between the potency of hallucinogens in the mouse HTR assay and their behavioral and subjective effects in humans was found [34]. Thus, the HTR dose–response is closely linked to 5‐HT2AR‐mediated behavior induced by psychedelic drugs in mice [53] as verified in mice lacking the 5‐HT2AR gene [6, 13]. In addition, 5‐HT2AR antagonists can block the HTR behavior induced by 5‐HT2AR agonists in rodents. Overall, the HTR is one of a few behavioral tests that can discriminate between hallucinogenic versus non‐hallucinogenic effects of 5‐HT2AR agonists and is a reliable test of drug efficacy (Table 2).

**TABLE 2:** Efficacy of 5‐HT2AR agonists and antagonists.

Effect properties					
Ligands	Agonism 5‐HT2AR	Effect	EC_50_/ED_50_	a (100%)	Model/behavioral tests
**5‐HT**	Full agonist	PI hydrolysis activation [36]	EC_50_ = 5.17 ± 0.97 nM at 5‐HT2AR [36]	= 100% at 5‐HT2AR [36]	Human embryonic kidney (HEK) 293 with high stable expression of wild‐type human 5‐HT2AR [36]
Psilocybin	Partial agonist	Calcium mobilization [39] HTR behaviors [39] Hypo‐locomotion [39]	Calcium mobilization: EC_50_ = 2132 nM [39] HTR behaviors: ED_50_ = 0.29 mg/kg SC [39] Hypo‐locomotion: ED_50_ = 2.8 mg/kg SC [39]	Calcium mobilization: 88% of 5‐HT activity [39]	Calcium mobilization: Cells transfected with human 5‐HT2AR [39] HTR behaviors: C57BL/6J male mice [39] Hypo‐locomotion: C57BL/6J male mice [39]
**Psilocin**	Full agonist Partial agonist Partial agonist	PI hydrolysis activation [36] 5‐HT2A activation [40] Calcium mobilization [39] HTR behaviors [39] Hypo‐locomotion [39]	EC_50_ = 7.29 ± 0.72 nM at 5‐HT2AR [36] EC_50_ = 0.721 ± 0.55 μM [40] Calcium mobilization: EC_50_ = 13 nM [39] HTR behaviors: 0.11 mg/kg [39] Hypo‐locomotion: ED50 = 1.8 mg/kg SC [39]	= 105 ± 9% of 5‐HT activity at 5‐HT2AR [36] = 16 ± 8% of 5‐HT activity [40] Calcium mobilization: 67% of 5‐HT activity [39]	Human embryonic kidney (HEK) 293 with high stable expression of wild‐type human 5‐HT2AR [36] Human 5‐HT2AR expressing mouse embryonic fibroblasts (NIH‐3T3 cells) [40] Calcium mobilization: Cells transfected with human 5‐HT2AR [39] HTR behaviors: C57BL/6J male mice [39] Hypo‐locomotion: C57BL/6J male mice [39]
Norpsilocin	Partial agonist	Calcium mobilization [39] HTR behaviors [39] Hypo‐locomotion [39]	Calcium mobilization: EC_50_ = 22 nM [39] HTR behaviors: ED_50_ > 30 mg/kg SC [39] Hypo‐locomotion: ED_50_ = 7.6 mg/kg SC [39]	Calcium mobilization = 89% of 5‐HT activity [39]	Calcium mobilization: Cells transfected with human 5‐HT2AR [39] HTR behaviors: C57BL/6J male mice [39] Hypo‐locomotion: C57BL/6J male mice [39]
**DOI**	Partial agonist	PI hydrolysis activation [36] Blocking 0.5 mg/kg MK‐801 neurotoxicity [54] HTR behaviors [55]	EC_50_ = 19.2 ± 2.6 nM at 5‐HT2AR [36] EC_50_ = 0.98 mg/kg [54]ED_50_ = 12.8 nmol/0.5 μL/brain side [55]	= 77 ± 3% (compared to 5‐HT) at 5‐HT2AR [36]	NIH‐3 T3 cells stably expressing rat 5‐HT2AR [36] Adult female Sprague–Dawley rats [54] Male Sprague–Dawley rats [55]
**Ketanserin**	Antagonist	—	—	—	—
**MDL 100, 907 = Volinanserin**	Antagonist	Antagonism of DOI‐induced HTR behaviors and ICSS depression [56]	AD_50_ = 0.0062 mg/kg to produce 50% antagonism of 1.0 mg/kg DOI‐ induced HTR behaviors [56] AD_50_ = 0.0040 mg/kg to produce 50% antagonism of 1.0 mg/kg DOI‐induced ICSS depression [56] AD_50_ = 0.00047 mg/kg to produce 50% antagonism of 0.32 mg/kg LSD‐ induced HTR behaviors [56]	—	HTR behaviors: C57BL/6 male mice [56] ICSS assays: Sprague–Dawley rats [56]

*Note:* Green = 5‐HT2AR agonist.; Orange = 5‐HT2AR antagonist.

Abbreviations: HTR = head‐twitch response behaviors, ICSS = intracranial self‐stimulation, PI = phosphatidylinositides.

A study in humans showed that pretreatment with the 5‐HT2AR antagonist ketanserin effectively prevented the psychedelic effects of psilocybin, establishing that engagement of the 5‐HT2AR is a key mode of action [57]. The psychedelic effects of psilocybin correlate with 5‐HT2AR occupancy and plasma psilocin levels [43]. Consequently, psilocin stimulation of cerebral 5‐HT2ARs is now considered a prime candidate molecular mechanism for psychedelic effects in the living human brain [43]. Thus, from the mid‐90s up to 2025, there is a consensus that 5‐HT2AR activation mediates the psychedelic effects of serotonergic psychedelics in humans and mice [6, 53]. However, the extent to which psilocin stimulates cerebral 5‐HT2ARs in the brains of humans and rodents and how this relates to its antidepressant effects has only recently been a focus of experimental study.

#### 3.1.3 Signaling Pathways of 5‐HT2AR: PLC, IP3 + DAG, β‐Arrestin and Internalization

Activation of 5‐HT2AR initiates a cascade of events leading to increased neuronal firing and activation of several intracellular signaling cascades. Briefly, the 5‐HT2AR is coupled to Gαq and β‐arrestin effector proteins. Serotonin is known to activate both 5‐HT2AR G_αi1_/G_αq/11_‐family G proteins and phospholipase C (PLC) signaling pathways, thus increasing post‐synaptic excitatory potentials in cortical pyramidal neurons and neurotransmitter release [58].

Sequestration of G protein‐coupled receptors (GPCR) from the cell surface is a commonly observed phenomenon following agonist stimulation. Initially characterized as desensitizing molecules, β‐arrestins 1 and 2 are important regulators of the endocytic process. The molecular mechanisms underlying the internalization of GPCR including 5‐HT2ARs have been carefully described by Robert Lefkowitz [59]. Thus, 5‐HT2ARs initiate cellular responses in post‐synaptic neurons and recruit β‐arrestin transducers that ultimately modulate GPCR activity.

#### 3.1.4 G‐Protein‐Biased 5‐HT2AR Agonists

Biased agonism is defined as the preferential activation of certain signaling pathways over others [60]. The hallucinogenic effects of psilocybin/psilocin at the 5‐HT2AR limit their clinical use. The synthesis of chemical analogs that retain therapeutic properties without causing hallucinations is currently underway to develop drugs that bias one of the downstream pathways of 5‐HT2A, i.e., PLC versus β‐arrestin2.

Serotonergic psychedelics effects of 5‐HT2AR agonists have been linked to a β‐arrestin‐dependent signaling pathways [8]. However, compared to other GPCRs, 5‐HT2AR can be internalized and desensitized in some cell types in a β‐arrestin‐independent manner [61]. G_αi1_ proteins are considered the effector pathway promoting hallucinogenic responses of psychedelics [62].

Lisuride is a preferential dopamine D2 receptor agonist that inhibits prolactin secretion and reduces hyperprolactinemia in patients with Parkinson's disease (PD) [63]. Because it directly stimulates post‐synaptic D2 receptor, its association with L‐DOPA, the precursor neurotransmitter to dopamine, strengthens synaptic dopaminergic functionality. Lisuride is also a G‐protein‐biased 5‐HT2AR agonist, but does not produce hallucinations in normal subjects at routine doses [64, 65].

βarr2‐biased 5‐HT2AR agonists were recently developed to know how the different signaling pathways contribute to the therapeutic effects mediated by serotonergic psychedelics [51, 66]. To gain molecular insights into psychedelic actions, Kim et al. [45] obtained the X‐ray crystal structures of 5‐HT2AR complexed with β‐arrestin‐biased ligands. Muneta‐Arrate et al. [48] demonstrated the biased inverse agonist profile towards 5‐HT2AR activation of G_αi1_ proteins of altanserin and pimavanserin, two antipsychotic drugs previously considered as selective 5‐HT2AR antagonists. These results strengthen the existence of functional selectivity of 5‐HT2AR for multiple G_αi1_/G_αq/11_‐protein pathways in neurons. Understanding the role of these 5‐HT2AR β‐arrestin‐1,2 signaling pathways in the antidepressant efficacy of psychedelics is crucial for a rational development of non‐psychedelic 5‐HT2AR agonists [65].

Some new compounds can activate brain circuits that help relieve depressive symptoms without triggering cellular pathways involved in hallucinations in mice. Cao et al. [51] suggest that the low transduction efficiency of structural analogs of LSD (0.0075 and 0.015 mg/kg, i.p. acutely in mice) can recruit β‐arrestin proteins to modulate GPCR activity. Such a cellular response of β‐arrestin–biased ligands of 5‐HT2AR, i.e., with a low transduction efficiency of 5‐HT2AR‐mediated signaling (i.e., β‐arrestin2 partial agonist), was associated with antidepressant‐like activity without hallucinogenic effects in the HTR. By contrast, psychoactive, hallucinogenic effects required a high transduction efficiency of 5‐HT2AR agonists at both G protein–mediated PLC signaling and β‐arrestin recruitment. Thus, these data suggest that β‐arrestin‐2 activity of 5‐HT2AR agonists plays a role in antidepressant‐like effects of serotonergic psychedelics but is not sufficient for inducing psychoactive actions [51]. This hypothesis is in line with a 50 to 70% 5‐HT2AR occupancy level required for an intense psilocybin‐induced psychological experience in humans [43].

The ability to promote cortical pyramidal neuron growth has been hypothesized to underlie the rapid and sustained therapeutic effects of psychedelics [67]. Surprisingly, using molecular and genetic tools, Vargas et al. [68] suggested that activation of an “intracellular population” of 5‐HT2AR is necessary for serotonergic psychedelics to mediate cortical structural plasticity and produce antidepressant‐like effects (however, only in the forced swimming test [FST]). The endogenous neurotransmitter serotonin did not display these properties. The finding that lipophilicity was a better predictor of psychoplastogenicity than 5‐HT2AR activation suggests that an intracellular pool of 5‐HT2ARs in cortical neurons might be responsible for psychedelic‐induced neuronal growth. This hypothesis needs to be further investigated to know whether intracellular 5‐HT2AR localization could be a future therapeutic target, and whether these effects extend past the FST for reversing depressive or anhedonic effects. To date, it is not known a priori whether these internalized receptors are still activatable or what their main signaling pathways are.

Vargas et al. [68] also studied 5‐HT2AR efficacy using Gq protein activation (using the traditional [3H]‐inositol phosphates accumulation assay) and β‐arrestin recruitment in rat embryonic cortical neurons treated with 5‐HT2AR ligands structurally related to serotonin. They emphasized the role of intracellular pools of 5‐HT2AR in cortical neuron growth, localized to a Golgi apparatus, a regulator of GPCR signaling. In agreement with Schmid and Bohn [69], this work suggests that HTR is a 5‐HT‐dependent response mediated by 5‐HT release by cortical pyramidal neurons in mice, which may result in circuit‐specific effects of psychedelics. Importantly, persistent cortical neuron growth and increased synaptic density after the drugs have been removed from the extracellular space is a hallmark not only of serotonergic psychedelics (LSD, psilocybin), but also of ketamine [70]. These long‐lasting structural remodeling in neuronal connectivity in the mPFC has been hypothesized to mediate their long‐lasting antidepressant effects of psilocybin via molecular mechanisms involving tyrosine receptor kinase B (TrkB), mammalian Target of rapamycin (mTOR) and α‐amino‐3‐hydroxy‐5‐methyl‐4‐isoxazolepropionic acid receptor (AMPA‐R) activation [71]. Future studies are necessary to confirm whether the intracellular population of 5‐HT2ARs contributes to neuroplasticity and hallucinogenic effects of psychedelics.

While these data are compelling, we caution the reader to note that conclusions of the above‐described studies are based only on the HTR, a behavioral *proxy* in rodents for human hallucinations induced by psychedelics. Additional behavioral tests to validate these findings are warranted. Environmental enrichment could be one of these tests coupled to HTR behavior [72]. By remodeling synaptic plasticity, enhanced environmental enrichment is known to increase adult hippocampal neurogenesis [73] and induce functional changes in the mouse cortical circuits [74]. The effects of context on the subjective experience induced by serotonergic psychedelics (psilocybin) have been recently investigated in mice: context and psilocybin indeed reorganized network architecture [75]. Social and communication disabilities are often reported in several major psychiatric disorders. Social communication involving social interactions associated with acoustic vocalizations has been measured in animal models. C57BL/6J mice are known to exhibit a higher rate of ultrasonic vocalizations during social interaction [76]. Thus, social behavior (social dominance, anxiety) in adult mice [77] could be added to a battery of behavioral tests studying psychedelic‐induced hallucinations in rodents.

Collectively, these findings suggest that many 5‐HT2AR agonists differentially activate PLC or β‐arrestin G_α_‐signaling pathways, stabilizing different active receptor states. When a certain signaling pathway is preferentially activated over the others, it could lead to varying qualities of efficacy for full, partial, inverse, or biased 5‐HT2AR agonists such as psychedelics [78].

Because biased signaling is easily identified and quantified in simple in vitro functional assays, pharmacological information from in vitro assays may not accurately translate to in vivo therapeutic systems. Nevertheless, preclinical research of biased 5‐HT2AR signaling has the potential to accelerate the discovery of more selective drugs for the treatment of a MDD and TRD, identifying safer non‐psychedelic 5‐HT2AR drug candidates devoid of hallucinogenic effects. Serotonergic psychedelics promote neuroplastic changes that may correct abnormal brain neural circuitry [79]. Overall, these data further highlight the gaps in knowledge that will need to be addressed to develop 5‐HT2AR ligands lacking hallucinogenic activity but with appreciable antidepressant‐like efficacy.

### 3.2 Brain Connectivity, Brain Imaging in Humans Versus Rodents

The development of our understanding of the neural mechanisms of psychedelics was stagnant for decades. However, functional imaging techniques have been set up mostly in the living human brain, with some studies that have ventured to image the rodent brain, reviewed in this section.

Positron emission tomography (PET) enables non‐invasive estimation of neurotransmitter changes and receptor occupancy in the living human brain [80]. PET is now applied to the study of psilocybin/psilocin. This imaging technique can reveal information regarding neural mechanisms of drugs and cerebral bioavailability underlying their therapeutic effects [81].

#### 3.2.1 In humans

Neuroimaging PET studies with 18F‐fluorodeoxyglucose (18F‐FDG) have demonstrated that serotonergic hallucinogens such as psilocybin increase frontal activity and induce a robust metabolic increase in the mPFC in 10 healthy volunteers [82]. In this clinical study of 18F‐labeled glucose analog uptake, changes in the rate of glucose metabolism likely correspond to changes in neurotransmitter function [82, 83].

Other tracers such as 11C‐Cimbi‐36, a potent 5‐HT2AR agonist without binding to dopamine or norepinephrine receptors, have been used in healthy volunteers. For instance, a PET scan study in healthy humans found that neocortex binding of 11C‐Cimbi‐36 to 5‐HT2AR predicts subjective effects of a single oral dose of psilocybin [84], representing a potential biomarker for efficacy that may be considered in future work.

Brain connectivity using fMRI investigated the effects of psilocybin before and after its administration in a small number (< 20) of healthy volunteers [25] or in patients with TRD [85]. David Nutt's team at the Imperial College London depicted correlates of psilocybin effects. Analysis of the map of cerebral blood flow after a single psilocybin injection (0.2 mg/mL perfused i.v., over 60 s, comparable with ∼15 mg of orally administered psilocybin, which is considered a moderate dose) revealed a decrease in the positive coupling between the mPFC and posterior cingulate cortex [25]. Correlation does not imply causation, and thus, we cannot definitively say that this cerebral blood signature induced by psilocybin directly led to therapeutic responses. However, these results suggest that both the subjective effects of psilocybin and its antidepressant effects are related to activity and connectivity changes in the brain's key connector hubs. A more recent fMRI analysis suggests that psilocybin relieves depressive symptoms through increasing connectivity between visual brain networks. Psilocybin‐induced decreases in sensitivity to neural inputs are associated with the perception of eyes‐closed visual imagery [86]. These fMRI studies suggest that the acute psychedelic effects of psilocybin may stem from drug‐level associated decreases in the duration of functional connectivity between lateral and medial frontoparietal regions also associated with plasma concentrations of psilocin and its subjective effects. Thus, the subjective effects of psilocybin may be caused by a decreased activity and connectivity in the brain's key connector hubs [87]. Yet, both fMRI and PET neuroimaging that included small cohorts of healthy individuals [88] found little changes in neural pathways mediating acute psilocybin effects at 1 week or 3 months post‐dose (0.2–0.3 mg/kg), indicating that there may be populations of individuals for which this drug is not efficacious.

However, a recent neuroimaging review reported alterations in the functional connectivity of psychedelics in healthy individuals, with consistent findings across substances of decreased connectivity within the default mode network and increased sensory and thalamocortical connectivity. Correlations between these neurophysiological changes and subjective experiences were noted, suggesting a brain network basis of the psychedelics' neuropsychological impact [89].

However, effects in healthy volunteers may not reflect what is going on in the brains of patients with depression. In addition, discrepancies between PET and fMRI studies, negative results, decreased or increased connectivity could be due to the small size of cohorts, differences in the dosage (< or > 1 mg/kg) and/or route of psilocybin administration (oral versus i.v.). Another possibility could be that PET and fMRI may involve different neurophysiological responses, e.g., activation of 5‐HT1A receptors (5‐HT1AR) by psilocin, the active metabolite of psilocybin [90]. More psychiatric neuroimaging studies need to be support clinical decision‐making. As Dunlop and Mayberg [91], recently wrote: “Circuitry‐based neuroimaging analyses can enhance our understanding of abnormal brain functioning in patients with MDD and other psychiatric conditions, but their utility for guiding treatment selection is less certain.” It will be important to fully report patient symptomatology, efficacious dose, and duration of improvement to further understand who can benefit most from receiving psilocybin treatment.

#### 3.2.2 In animals

There are very few studies that we are aware of that have used imaging in animal models to uncover the effects of psilocybin. One study described the effects of a single dose of psilocybin (0.08 mg/kg i.v.) on 5‐HT2AR occupancy in the pig brain [92].

In rodents, in vivo PET imaging studies on psilocybin/psilocin in brain regions are sparse. Various PET radioligands such as 5‐HT2AR agonists (^11^C‐Cimbi‐36 [93]) and a 5‐HT2AR antagonist (^11^C‐M100907 [94]) and the selective 5‐HT2AR radioligand, ^18^F‐altanserin, also a substrate of P‐glycoprotein [95], were used to quantify 5‐HT2AR densities in vivo in the rat brain. Psychedelics such as DOI dysregulated cortical activity, producing an enhanced global connectivity [96]. Separately, using fMRI in anesthetized rats, Artigas and colleagues confirmed that the natural hallucinogen 5‐MeO‐DMT, a component of ayahuasca, disrupted cortical function [97]. Extended information was obtained using local‐field potential recordings of pyramidal neurons in freely moving mice [98]: 5‐MeO‐DMT alterations of cortico‐thalamic networks were connected to its antidepressant‐like effects. It is well known that the activation of 5‐HT2AR in the mPFC by the hallucinogen DOI increases the firing activity of the DRN 5‐HT neurons and cortical 5‐HT release. Thus, the Artigas group also showed that DOI increased the activity of the mPFC‐DRN circuit by acting on postsynaptic 5‐HT2AR unrelated to thalamocortical afferents [99].

Recently, the acute effects of a single administration of psilocybin (0.5–20 mg/kg) on the expression of selected genes and corresponding protein levels were assessed in rat brain tissue [100]. In the mPFC, psilocybin increased gene expression related to neuroplasticity, e.g., immediate early genes c‐fos, FosB, Junb. A single dose of psilocybin (2 mg/kg) also increased c‐Fos expression in various brain regions such as the neocortex, striatum, amygdala in mice [75]. Taken together, these results suggest that psilocybin can induce a rapid reorganization of the brain network architecture.

By contrast, brain imaging studies have extensively described the psychoactive, dissociative effects of (*R,S*)‐ketamine, the fast‐acting glutamatergic antidepressant [9]. For example, brain glucose metabolism was recently studied using [^18^F]‐FDG PET imaging in rats after an acute sub‐anesthetic dose of ketamine (10 mg/kg) [81]. Glucose uptake was decreased 5 days post‐dose, reflecting a sustained and delayed effect of ketamine in the frontal and cingulate cortex. An increase in the raphe, caudate, and cerebellum was also measured. Moreover, metabolic connectivity analyses revealed a decreased connectivity between the hippocampus (HP) and the thalamus at day 5 compared to the baseline. Note that the psychodysleptic effects of ketamine diverge from those of serotonergic psychedelics such as psilocybin, with a special “hallucinatory” dimension. Unlike serotonergic 5‐HT2AR psychedelics, NMDA receptor (NMDA‐R) antagonists such as ketamine display dissociative symptoms. Psychotomimetic symptoms can occur within an hour following the administration of both ketamine and psilocybin [2].

Thus, neuroimaging studies using PET scan, fMRI in patients with depression treated with psychedelics are still sparse: further preclinical and clinical investigations are requested to clarify the benefits of brain imaging for each sub‐population of MDD.

### 3.3 Pharmacokinetics of Psilocybin/Psilocin in Rodents

The metabolism of the prodrug psilocybin is similar in humans and rodents, i.e., a rapid dephosphorylation in the intestine and liver to form psilocin (4‐hydroxy‐*N,N*‐dimethyltryptamine), its active metabolite contributing to the neurobiological effects via a 5‐HT2AR activation. Importantly, psilocybin cannot cross the blood–brain barrier, unlike psilocin [101] which has a more lipophilic property that allows its brain delivery [102].

After an oral dose in rodents, psilocin is distributed to all tissues, including the brain, and is excreted within 24 h: its plasma elimination half‐life t1/2 = 117 min in rodents [103, 104]. Psilocybin and its metabolites are mainly excreted in the urine in 24 h [105]. Psilocin undergoes glucuronidation or conversion to norpsilocin (4‐hydroxy‐*N*‐methyltryptamine), which produces HTR in a dose‐responsive manner in mice [106], but no cognitive effects in rats [107]. Relevant interactions at 5‐HT receptors remain to be determined [108].

Of note, after a single oral dose in humans (healthy volunteers), the plasma elimination half‐life t1/2 is 50 min and 2.5 h for psilocybin and psilocin, respectively [104, 109, 110]. The psychodysleptic effects begin approximately 1 h after oral administration of psilocybin and continue for 4 h [90]. A close positive association of plasma psilocin levels with a cerebral 5‐HT2AR occupancy was found in a PET scan study performed for the first time in the living human brain after a single dose of psilocybin given to healthy volunteers [43].

In human liver microsomes, CYP2D6 and CYP3A4 enzymes metabolized nearly 100% and 40% of psilocin, respectively [108]. These findings are interesting for studying drug–drug interactions of psilocybin as a fast‐acting antidepressant drug.

## 4 Antidepressant‐Like Effects of Psilocybin and Psilocin in Rodents

A key question to address in the field, which also happens to be an active area of debate, is whether the immediate subjective effects of psychedelics are necessary to produce their long‐lasting therapeutic responses in patients with depression [6, 14, 111]. Recent experimental publications in rodents suggest that the hallucinogenic effects of psychedelics can be separated from their antidepressant‐like and fronto‐cortical neuroplasticity effects. Olson's team modified a psychedelic compound (ibogaine, tabernanthalog, lisuride) to produce a non‐hallucinogenic variant that has antidepressant‐like effects in rodents mild stress models (but does not seem to produce HTR [51, 112]). Some neuroscientists presumed that their antidepressant actions require altered consciousness, which is known to be dependent on 5‐HT2AR activation [15], but others disagree [6, 14]. Here, we selected three main reports addressing the question regarding the role of 5‐HT2AR activation in the antidepressant‐like effects of serotonergic psychedelics. These are recent publications with complementary experimental approaches: a pharmacology protocol alone [14], a genetic protocol alone [6], and a protocol combining pharmacology and genetics [15]. Their conclusions diverge.

In a restraint stress model of anxiety/depression (immobilization 4 h/day for 10 to 14 consecutive days), a single injection of psilocybin (1 mg/kg, i.p.) reversed anhedonic responses assessed in behavioral tests, e.g., sucrose preference, HTR behavior [14]. This anti‐anhedonic response to psilocybin was associated with a strengthening of excitatory synapses in the HP, a characteristic of fast‐acting antidepressant drugs such as ketamine. Neither behavioral nor electrophysiological responses to psilocybin were prevented by pretreatment with ketanserin, a 5‐HT2A/2C receptor antagonist. The authors concluded on a 5‐HT2AR‐independent antidepressant‐like and synaptic effects of psilocybin in C57BL/6J mice that lacked the canonical hallucinogenic features of rodent psychedelic response.

However, some points need to be discussed regarding the pharmacological approach used in this study:

- The timing and the dose of ketanserin administration (2 mg/kg, a high dose administered 60 min prior to psilocybin) must be carefully investigated before concluding that there is no blockade of psychedelics' effects by an antagonist.
- A more selective 5‐HT2AR antagonist, M100907, should be tested to more convincingly conclude that this is the mechanism [113].
- Behavioral effects of psilocybin were measured 24 h after its systemic administration: both behavioral and neurochemical effects should be investigated at several time points post‐injection (e.g., 2, 12, 48 h, and 1 or 2 weeks) to determine long‐lasting effects of a single dose.
- In vivo electrophysiology performed in male C57BL/6J mice: a huge acute psilocybin dose (10 mg/kg, i.p.,) was used to measure hippocampal local field potential activity.

Similarly, Sekssaoui et al. [6] investigated the antidepressant‐like effects of two psychedelics of different chemical families, DOI and psilocybin, and a non‐hallucinogenic 5‐HT2AR agonist, lisuride, in a mouse model exhibiting a depressive‐like phenotype (chronic behavioral despair model, CDM). A single administration of each drug (each at 1 mg/kg, i.p.) induced an antidepressant‐like effects in wild‐type C57BL/6J mice in several behavioral tests 48 h and 11 days post‐administration. By contrast, DOI and lisuride administration did not produce antidepressant‐like effects in 5‐HT2AR knock‐out mice, whereas psilocybin was still effective. Thus, these findings suggest that 5‐HT2AR agonists can produce antidepressant‐like effects independently of hallucinogenic properties through mechanisms that may or may not be via the 5‐HT2AR. For the genetic approach, a “constitutive” 5‐HT2AR knock‐out (KO) line of mice from René Hen and Jay Gingrich labs at Columbia [13] was used. Compensatory responses to the constitutive deletion of the 5‐HT2AR in these adult KO mice may have occurred during the development of the brain [114]. To prevent this possibility, future studies using “inducible, tissue specific” 5‐HT2A KO mice are needed to confirm these results. Collectively, these two studies performed in mouse models of anxiety/depression support the idea that subjective effects of psychedelics may not be necessary to produce long‐lasting therapeutic benefits in humans [111].

By contrast, Cameron et al. [15] reached a disparate conclusion by using a combination of pharmacological and genetic approaches. Activation of 5‐HT2AR is essential for tryptamine‐based psychedelics to produce antidepressant‐like effects in CORT‐induced anhedonia in rodents. In this model, psychedelic tryptamines such as psilocybin and 5‐MeO‐DMT (each at 10 mg/kg, i.p.) induced hallucinogenic and therapeutic effects 24 h post‐administration, through activation of the same 5‐HT2AR as assessed 24 h post‐dose, since these effects were blocked by ketanserin (4 mg/kg, i.p.) and absent in 5‐HT2AR KO mice (129S6/SvEv background). Some points can be discussed about this study:

- Dendritic spine density was assessed 24 h after administration of 5‐MeO‐DMT compared to ketamine (each at 10 μM) in rat embryonic cortical culture. This experiment confirmed that 5‐MeO‐DMT can promote synaptogenesis as well as DMT, psilocin [70] and ketamine (here and in Li et al. [19]).
- Ex vivo electrophysiology: mice were anesthetized with isoflurane, then local field potentials were recorded in cortical neuronal slices 24 h after 10 mg/kg, i.p. psilocybin injection. The dose is relatively high as in behavioral tests.
- The sucrose preference test was performed 24 h post‐5‐MeO‐DMT treatment in CORT pre‐treated mice, but FST and HTR responses were measured in naïve mice treated with psilocybin. The FST is a screening test used to predict antidepressant drug potential, rather than a model of stress. It does not induce a depressive phenotype [115].

This study agrees with Yaden and Griffiths [116] saying that some subjective effects occasioned by moderate to high doses of psychedelics in humans are necessary for their full and enduring therapeutic effects.

These different results described above in these three reports could be explained by differences in protocols. Notably, the genetic approach is interesting for supporting the results of pharmacological studies. The strain of 5‐HT2AR KO mice, originally generated on a 129S6/SvEv background and backcrossed over at least 10 generations onto the inbred C57BL/6J line [6] versus the 129S6/SvEv background [15]. The sucrose preference test was performed in a stress model of anxiety/depression, the CORT‐induced anhedonia model (10 days of corticosterone treatment, daily i.p. injection of 20 mg/kg) [15]. It should be interesting to test for different doses of psilocybin/psilocin in rodent models of anxiety/depression [117], instead of a single dose. As shown in Table 3, experimental protocols performed in rats and mice are heterogeneous.

**TABLE 3:** Antidepressant‐like effects of psilocybin/psilocin in rodents.

	Dose (mg/kg), route	Rodents	Time of study	Results	References
Psilocin	0.3–4.8, s.c.	WT: C57BL/6J male 5‐HT2AR KO mice	Immediately post‐dose	Head‐twitch, Mouse behavioral pattern monitor, locomotion	Halberstadt et al., 2011 [118]
Psilocin	1–5‐ 10, i.p.	Male Wistar rat	Immediately post‐dose	μD 5‐HT, DA in VTA, Nacc, mPFC HTR	Sakashita et al., 2015 [119]
Psilocin	0.03–2.0, i.v.	Male Sprague–Dawley rats	Immediately post‐dose	Neuro‐vascular, imaging (fMRI)	Spain et al., 2015 [120]
Psilocin	0.25–1‐ 4, s.c.	Wistar Rat	15 min post‐dose	Prepulse inhibition of the acoustic startle reaction, Open field (OF)	Tyls et al., 2016 [121]
Psilocin and ketamine	0.05–0.075, s.c. 0.05–3	Male Wistar rats	48 h after microdosing (6 days)	Elevated plus maze (EPM)	Horsley et al., 2018 [122]
Psilocin psilocybin	0.5–2, i.p. 3–10	Male Flinders Line rats (Sensitive and resistant)	24 h post‐dose (E1 and E2), 8 days (E3), 4 h post‐dose (E4)	FST, OF	Jefsen et al., 2019 [117]
Psilocin, norpsilocin Psilocybin (R)‐ketamine (S)‐ketamine	0.22–0.72, i.p. 0.3, 1 7.5, 15, 30, i.p. 3.5, 7.5, 15	Sprague–Dawley Rat	Immediately post‐dose	Cognition	Popik et al., 2022 [107]
Psilocybin Ketamine LSD	1, i.p. 5, 20, 100, i.p. 0.15	Sprague–Dawley Rat	7, 14, 21, 28, 35 days post‐dose (FST) 41 days post‐dose (EPM)	FST, EPM	Hibicke et al., 2020 [123]
Psilocybin Ketanserine	1, i.p. 2, i.p.	Chronic multimodal stress paradigm (CMMS) C57BL/6 J mice	24 h post‐dose	HTR, FST, sucrose, hippocampal slice electrophysiology	Hesselgrave et al., 2021 [14]
Psilocybin Ketamine	2 * ‐ 10 10, i.p. *Dose active	Rat	Immediately post‐dose 24 h post‐dose	μD 5‐HT, DA, Glu, GABA in the mPFC. OF, Light Dark Box, FST	Wojtas et al., 2022 [124]
Psilocybin Ketamine Ketanserin 5‐MeO	10 3 1–4 10	Rat WT C57/BL6J, KO 5‐HT2AR mice (129S6/SvEv)	24 h post‐dose 30 min, Day1 to D14 ‐Immediately −30 min, 24 h, D7	Embryonic cortical cell culture CORT model ‐HTR ‐FST, sucrose and electrophysiology	Cameron et al., 2023 [15]
Psilocybin	2	(TRAP2, Ai14) mice 25 males, 8 females	2 h post‐dose	Environmental context, c‐fos immunofluorescence, iDISCO cleared tissue	Rijsketic et al., 2023 [75]
Psilocin DOI	1.5, 2 and 4 0.05, 0.1, 0.25, 1.0, and 2.0	Male C57BL/6 J mice	24 h post‐dose	FST, TST, NSF, three‐chamber sociability test, OF, HTR	Takaba et al., 2024 [125]
Psilocybin, DOI Lisuride Ketamine	1, microdosing 0.05; i.p. 1 3	CDM male and female C57BL/6 J mice 5‐HT2AR KO mice	NSF 48 h SP Day 11 FST Day 15	FST, sucrose preference, NSF	Sekssaoui et al., 2024 [6]

Amazingly, in a phase 2 clinical trial in patients with TRD, a single dose of psilocybin can relieve symptoms of depression over several weeks [5]. Such long‐lasting effect of psychedelics has been attributed to their ability to modify addiction‐related neural circuitry through their activation of neurotrophic factor signaling [112].

### 4.1 Remarks on other current issues

The positive effects of microdosing psychedelic intake on mood state and cognitive processes are still being uncovered. There are very few clinical studies that have specifically addressed this issue, none of them being double‐blind, placebo‐controlled clinical trials of psychedelics microdosing [111]. Microdose usage is supported by preclinical studies showing that acute administration of very low doses of LSD (0.015 mg/kg, i.p.) induced an antidepressant‐like effect, but failed to induce HTR activity in mice [51]. These data suggest that the hallucinogenic effect may not be required for the antidepressant‐like effect of LSD or DMT [126]. Further preclinical studies need to be conducted to shed light on the potential microdosing consequences. Recently, DOI and psilocybin were injected daily for 6 days at the dose of 0.05 mg/kg that does not induce HTR in wild‐type mice [6]. This daily microdosing protocol was as efficient as a single dose administration to induce antidepressant‐like effects in stressed mice [6]. One of the advantages of microdosing clinical trials compared to those using high doses of psychedelics would be to effectively blind both patients with depression and clinicians. However, a question remains: will low doses of serotonergic psychedelics required to avoid significant subjective effects be insufficient to activate 5‐HT2AR to produce long‐lasting changes in neural circuitry [111]?

In addition, the intra‐nasal route of administration with a direct access to the brain [18] should be investigated as it was done for esketamine. Furthermore, (±)‐ketamine, as a fast‐acting antidepressant drug, should be a reference compound in preclinical [6] and clinical studies [22] (Tables 4A and 4B).

**TABLE 4A:** Comparison of psilocybin/psilocin versus ketamine: clinical studies.

	Psilocybin/Psilocin	Ketamine, Esketamine	References
Duration of clinical effects	One or two doses persistently mitigate the symptoms of MDD and anxiety for 3 weeks or 3 months	A single sub‐anesthetic dose: antidepressant effects peaked at 24 h post‐infusion and faded after 10–12 days	Hinkle et al. (2024) [33] Goodwin et al. (2022) [5] Carhart‐Harris et al. (2016) [3] Thomann et al. (2024) [108]
Stupefying effects	Psychedelic and psychotomimetic states (transient paranoïa), visual and auditive hallucinations, euphoria/anxiety, but no dissociative effects. No addiction!	Transient psychotomimetic and dissociative states	Johnston et al. (2023) [10]
Drug agencies, USA, Europe	FDA designated psilocybin as a breakthrough therapy in 2018 for TRD and in 2019 for MDD	FDA and EMA approved Esketamine SPRAVATO nasal spray, for adults with TRD (2019), for adults with MDD and suicidal ideation (2020)	https://www.fda.gov/ https://www.ema.europa.eu/ https://ansm.sante.fr/

**TABLE 4B:** Comparison of psilocybin/psilocin versus ketamine: Preclinical studies.

	Psilocybin/Psilocin	Ketamine, Esketamine	References
Pharmacological property	Psilocybin, a pro‐drug, inactive Dephosphorylation Psilocin, metabotropic GPCR serotonin 5‐HT2A, receptor agonist, active molecule, lipophilic, cross BBB, frontal cortex spines density in pyramidal neurons	‐ Ionotropic NMDA glutamate receptor antagonist—Synaptogenesis in the mPFC ‐ Fast (24 h) and sustained (1 week) antidepressant effects after an acute administration	Duman et al., (2016) [127] Shao et al., (2021) [70] Revenga et al., (2021) [128] Hesselgrave et al., (2021) [14] Sekssaoui et al., (2024) [6]
Liver metabolism	Psilocin: CYP2 D6 100%, CYP4A4 40% No gene polymorphism 4‐HIAA inactive molecule	CYP 2D6, CYP3A4, CYP2C9/2C19; SNIP genetic polymorphism (e.g., BDNF Val66Met), drug interactions Controversy: (2R,6R)‐hydroxy‐norketamine (6)‐HNKs, active metabolite(s)?	Zanos et al., 2016 [129] Thomann et al. (2024) [108]
PK in rodents	t_1/2_ ≤2 h, route of administration: per os	t_1/2_ ≤2 h, route of administration: intravenous or per os LP	Glue et al., 2024 [130] Thomann et al. (2024) [108]

The effects of context on the subjective experience of serotonergic psychedelics have not been examined in rodents yet, due to technical limitations of behavioral tests. However, Rijsketic et al. [75] attempted to tackle this question by examining the impact of an enriched environmental context on psilocybin‐elicited neural activity at cellular resolution. c‐Fos‐immunofluorescence revealed clusters of neural activity changes in several brain regions (c‐Fos expression increased in the *cortex*, *amygdala*, but decreased *in the hippocampus*, *striatum*) associated with main effects of context and psilocybin treatment. Psilocybin decreased or increased c‐Fos^+^ cell density in various brain regions in a predominantly context‐independent manner, but main effects of context and psilocybin were robust, widespread, and reorganized network architecture. Although an enriched environment is known to activate adult hippocampal neurogenesis [73], efforts such as this one (*administering psilocybin to mice in their home cage or an enriched environment*) to translate the subjective experience of serotonergic psychedelics observed in humans to animal models must be continued (*not in naïve, unstressed mice*). Indeed, reduced spine density and decreased neurotransmission occur in the frontal cortex of rodent chronic stress models [131], as in patients with depression [132].

## 5 Effects of Psilocybin and Psilocin on Brain Neurotransmitter Systems

Psilocybin exerts its effects by activating 5‐HT2AR, primarily located on cortical pyramidal neurons in regions such as the mPFC and HP. There is evidence in rats suggesting that activation of 5‐HT2AR enhances neurotransmitter release from a similar subset of glutamatergic terminals that innervate the apical dendrites of layer V pyramidal cells [133, 134]. A study investigated the comparative effects of psilocybin and ketamine on brain neurotransmitter systems in rats (Table 4C). Psilocybin induced dose‐dependent increases in the extracellular levels of serotonin (5‐HT_ext_), dopamine (DA_ext_), glutamate (GLU_ext_), and GABA (GABA_ext_) as measured by microdialysis, while ketamine exerted a more pronounced effect on cortical 5‐HT_ext_ and DA_ext_ with a modest impact on GLU_ext_ [124]. Likewise, another study evaluating the effects of psilocin on dopamine and 5‐HT transmission in the ventral tegmental area (VTA), nucleus accumbens, and mPFC showed that psilocin mainly increases DA and 5‐HT levels in the meso‐accumbens and/or meso‐cortical pathways [119]. This modulates excitatory transmission and stimulates GABAergic interneurons. In addition, DOI, another hallucinogenic 5‐HT2AR agonist, increases cortical Glu ext (i.e., glutamate release) [138]. 5‐HT2AR are colocalized with 5‐HT1AR on GABAergic interneurons: psilocybin has a lower affinity for 5‐HT1AR than for 5‐HT2AR [135]. The balance between excitatory and inhibitory neurotransmission, mediated by interactions between 5‐HT2A‐R and 5‐HT1A‐R, plays a critical role in psilocybin's subjective effects in humans, such as “ego dissolution” [136]. Increased GLU_ext_ in the mPFC correlates with negative ego dissolution experiences, whereas reduced GLU_ext_ in the HP is linked to positive experiences [136]. These findings underscore the importance of understanding the GLU/GABA balance, receptor interactions in brain circuits, and their expression to optimize therapeutic doses of psilocybin while minimizing adverse effects such as “bad trips.” Future preclinical research aims to refine these mechanisms to enhance its efficacy.

**TABLE 4C:** Comparison of psilocin versus ketamine: Brain modifications.

	Psilocybin/Psilocin	Ketamine	References
Neurotransmitters effects	Increases 5‐HT, DA, GLU, and GABA levels; dose‐dependent.	Stronger effect on DA and 5‐HT; modest GLU impact	Wojtas et al, 2022 [124]
Mechanism of action	Directly increases pyramidal neuron excitability and glutamate release.	Reduces inhibition by suppressing GABAergic signaling, indirectly increasing pyramidal neuron activity.	Shao et al, 2021 [70] Szpregiel and Bysiek 2024 [135] Mason et al, 2020 [136]
Neuroplasticity	Induces long‐lasting dendritic spine growth and synaptic remodeling via AMPA and NMDA receptors	Promotes transient dendritic spine growth and synaptic remodeling via AMPA activation	Szpregiel and Bysiek 2024 [135] Wojtas et al, 2022 [124] Shao et al, 2021 [70] Moda‐Sava et al., 2019 [134]
Glutamate Pathway	Involves GluN2A subunits on glutamatergic neurons and early response genes like c‐Fos	Modulates glutamate release through GluN2B subunits on GABAergic interneurons	Wojtas et al, 2022 [124] Davoudian et al, 2023 [137]

Moreover, structural neuroplasticity in the frontal cortex plays a crucial role in the effectiveness of antidepressant drugs [132]. Mood disorders are associated with synaptic atrophy mainly in the mPFC, as well as structural deficiencies such as reduced dendritic branching, lower spine density, and impaired neurotransmission, which have been observed in rodent models of chronic stress. In contrast, fast‐acting antidepressants work by enhancing structural plasticity, helping to restore the synaptic connections damaged by chronic stress [139]. Psilocybin promotes neuroplasticity in the mPFC through its influence on glutamatergic signaling and serotonin receptor activation, particularly 5‐HT2A‐R. The increase in GLU_ext_ stimulates the expression of brain‐derived neurotrophic factor (BDNF) [140]. The increased BDNF expression and GLU release by psilocybin likely lead to the activation of postsynaptic AMPA‐R in the mPFC and, consequently, increased neuroplasticity [135]. These effects, essential for neuroplasticity, occur independently of 5‐HT2A‐R activation, separating psilocin's neuroplastic and hallucinogenic properties. However, most conclusions are based on in vitro and molecular docking studies, which, while robust, may not fully reflect in vivo dynamics. Validation in animal models or human clinical trials is essential to confirm the relevance of these findings in real‐world contexts.

GluN2A/2B containing NMDARs specifically in pyramidal neurons may also be required for fast antidepressant‐like effects and for regulation of protein synthesis involved in synaptogenesis. It was already described for ketamine, i.e., GluN2B antagonists display the same antidepressant‐like effects as ketamine. Similar investigations are still requested for psychedelics. Psilocybin also influences NMDA‐R subtypes, specifically by increasing the expression of the GluN2A subunit 24 h after administration of 10 mg/kg in rats [124]. This may contribute to early neuroplastic changes alongside activation of immediate early genes' response like c‐Fos, which regulates neuronal adaptation after an acute administration of either ketamine, psilocybin, or 5‐HT2AR agonists [137]. Thus, synaptic rewiring could be a common mechanism underlying the rapid antidepressant effects of psilocybin. Interestingly, the impact of psilocybin on neural architecture resembles that of ketamine, which, at subanesthetic doses, similarly induces a rapid increase in spine density and an elevated rate of spine formation in the mPFC [141, 142]. Furthermore, changes in dendritic spines after psilocybin are persistent for at least a month, unlike ketamine, which produces a sustained (24 h) antidepressant effect [70, 135]. The interplay of excitatory and inhibitory effects in both substances allows a selective synaptic plasticity [22, 143].

Psilocybin also enhances the release of acetylcholine (ACh), particularly in the HP, which is crucial for synaptic plasticity and cognitive processes such as learning and memory [144]. This effect is mediated by the activation of serotonin 5‐HT2AR, which increases glutamate release and indirectly stimulates ACh production. Additionally, 5‐HT1AR, also targeted by psilocybin, may contribute to ACh release, creating a synergistic interaction between these serotonin receptor subtypes. By enhancing ACh signaling, psilocybin may support neuronal communication and cognitive flexibility, offering potential benefits for neuropsychiatric conditions that involve deficits in cholinergic function [135].

Although there is no formal evidence on its antidepressant mechanism, psilocybin/psilocin could modulate cortical–limbic connectivity and regulate the functional alterations found in depression [2]. It reduces the hyperactivity of the mPFC observed in mood disorders [25], with a positive correlation between these effects measured in functional imaging and its clinical effectiveness on mood. Its impact on glutamatergic signaling via mGluR2–3 glutamate receptors could also promote neuroplasticity via an activation of BDNF/TrkB/mTOR signaling cascade, as ketamine [22, 30, 70].

## 6 Psychedelics, Ketamine, the Neurotrophic Factor BDNF and Its High‐Affinity TrkB Receptor

BDNF has been implicated in the physiopathology of MDD and antidepressant drug action. It has fundamental functions in synaptic plasticity in the adult brain following antidepressant drug treatment. Increased expression and signaling of BDNF have been repeatedly implicated in the mechanism of the fast‐acting antidepressant drugs, mainly ketamine, but also psilocybin.

The role of single nucleotide polymorphisms (SNP), such as the Val66Met (e.g., rs6265), a functional SNP within the first exon of BDNF affecting ketamine response, was studied in patients with MDD: those with the Val/Val BDNF allele at rs6265 are more likely to exhibit increased antidepressant response to ketamine than Met carriers [145]. However, homozygous Met BDNF carriers are rare (< 5%) in the European‐ancestry population, compared to homozygous Val/Val individuals (≈60%) (both Val/Met and Met/Met alleles). Plasma BDNF levels in Caucasian adult patients with MDD were linearly associated with the BDNF Val66Met genotypes, Met carriers having lower BDNF levels than ValVal ones [146]. Su and Krystal [147] characterized the dose‐related efficacy of ketamine in TRD and its effects in a genotyped Chinese population in which most (83%) patients possessed at least one copy of the lower functioning Met allele of the BDNF gene. However, among clinical studies on ketamine, results are mixed. Laje et al. [148] suggest that an increased BDNF function is a necessary component of the antidepressant response of ketamine and other NMDA‐R antagonists. By contrast, Smit et al. [145] are skeptical, claiming that recent data do not support sufficient reliability of using serum/plasma BDNF levels to characterize depression or to predict antidepressant response in clinical use. Brain BDNF is likely involved in MDD pathophysiology and antidepressant drugs' efficacy, but its reflection in peripheral BDNF is of limited diagnostic or prognostic utility [145].

To date, no clinical studies described a role of BDNF Val66Met genetic polymorphism SNP in psilocybin antidepressant response in patients with MDD or TRD. Thus, more research is needed to elucidate the role of BDNF/TrkB receptor (TrkB) signaling in the sustained antidepressant response of Caucasian and Asian patients with TRD.

Regarding preclinical studies with ketamine, results depend on the strategy used to study the role of BDNF/TrkB in its fast‐antidepressant‐like effects. TrkB receptor (neuronal receptor tyrosine kinase‐2, NTRK2‐TrkB), the high affinity receptor for BDNF, is a central regulator of activity‐dependent neuronal plasticity [149]. A single subanesthetic dose of ketamine induced similar antidepressant‐like responses in wildtype (WT) and BDNF^+/−^ heterozygous mice and did not influence BDNF levels or TrkB phosphorylation in the HP when assessed at 45 min or 7 days post‐dose [150]. Thus, these authors claimed that unlike SSRI/SNRI, BDNF signaling does not play a pivotal role in the antidepressant effects of glutamate‐based compounds.

However, recently, more complete *in silico* and in vitro studies from the same Eero Castren's laboratory challenged this conclusion. They studied susceptibility to chronic stress and antidepressant treatment response to fluoxetine and ketamine [151] as well as psychedelics such as LSD and psilocybin [67]. They proposed a direct binding of both classical monoaminergic SSRI‐SNRI and fast‐acting antidepressants to TrkB, thus facilitating BDNF action and plasticity and accounting for cellular and behavioral effects (HTR, freezing) of these drugs.

In a second study, Eero Castren's group used the same *in silico* and in vitro tests showing that both psychedelics, LSD and psilocin, bind with a nanomolar affinity to TrkB (Ki = 6.73 ± 3.05 nM in HEK293T cells) and the binding is impaired in TrkB‐R Y433F^+/−^ mutant mice [67]. This mutation blocks the induction of neuroplasticity and long‐term plasticity responses but does not affect the HTR (*an LSD‐like behavioral profile in rodents)*. Moreover, 5‐HT2AR antagonists fail to prevent psychedelic‐induced TrkB dimerization and neurotrophic signaling, spino‐, dendrito‐genesis, and antidepressant‐like behavioral effects. In a rodent model of chronic stress consisting of repeated sessions of the FST, they found a sustained antidepressant‐like effect 7 days after a single LSD administration in WT but not Y433F^+/−^ mutant mice [67].

Collectively, both a pharmacological blockade and a genetic TrkB mutation, mainly in vitro experiments suggest that neuroplasticity induced by psychedelics (*TrkB dimerization, activation of synaptogenesis, and dendritic arborization)* and their antidepressant‐like activity in mice depend on binding to TrkB and activation of BDNF signaling, but are independent of 5‐HT2AR activation. By contrast, HTR depends on 5‐HT2AR activation and is independent of the BDNF/TrkB signaling pathway in neuronal culture [67].

Overall, this set of behavioral data is not convincing. To avoid misunderstanding and misestimated effects of drugs in the FST, it is necessary to do the following:

- to perform more than one test of the antidepressant‐like action of the molecule in rodents;
- despite the assertion of Vestring et al. [152], the FST is not a chronic despair model. The test was originally set up by Porsolt et al. [153] to identify the antidepressant potential of drugs in a time when the understanding of behavioral effects of antidepressant drugs were still in their infancy.. The immobility response anthropomorphically is not a measure for depression and despair. It shows a switch from active to passive behavior in the face of an acute stressor, aligned to cognitive functions underlying behavioral adaptation and survival [154]. More importantly, there is no consensus about this hypothesis for how ketamine and psilocybin/psilocin exert their fast‐acting antidepressant activity by direct binding to TrkB, as a common mechanism of action for SSRI/SNRI as well as fast‐acting antidepressant drugs [155]. Overall, more basic and clinical research is needed to specify the antidepressant activity of psychedelics such as psilocybin/psilocin.

## 7 Perspectives and Conclusion

Serotonergic psychedelic compounds and ketamine can produce a rapid and long‐lasting antidepressant effects engaging plasticity mechanisms (synaptogenesis) after a single administration. They represent a new era in the development of antidepressant drugs focused on promoting the growth of cortical pyramidal neurons and fixing neural circuits rather than rectifying chemical imbalances as described for conventional serotonergic/noradrenergic treatments [111].

**FIGURE 1:**
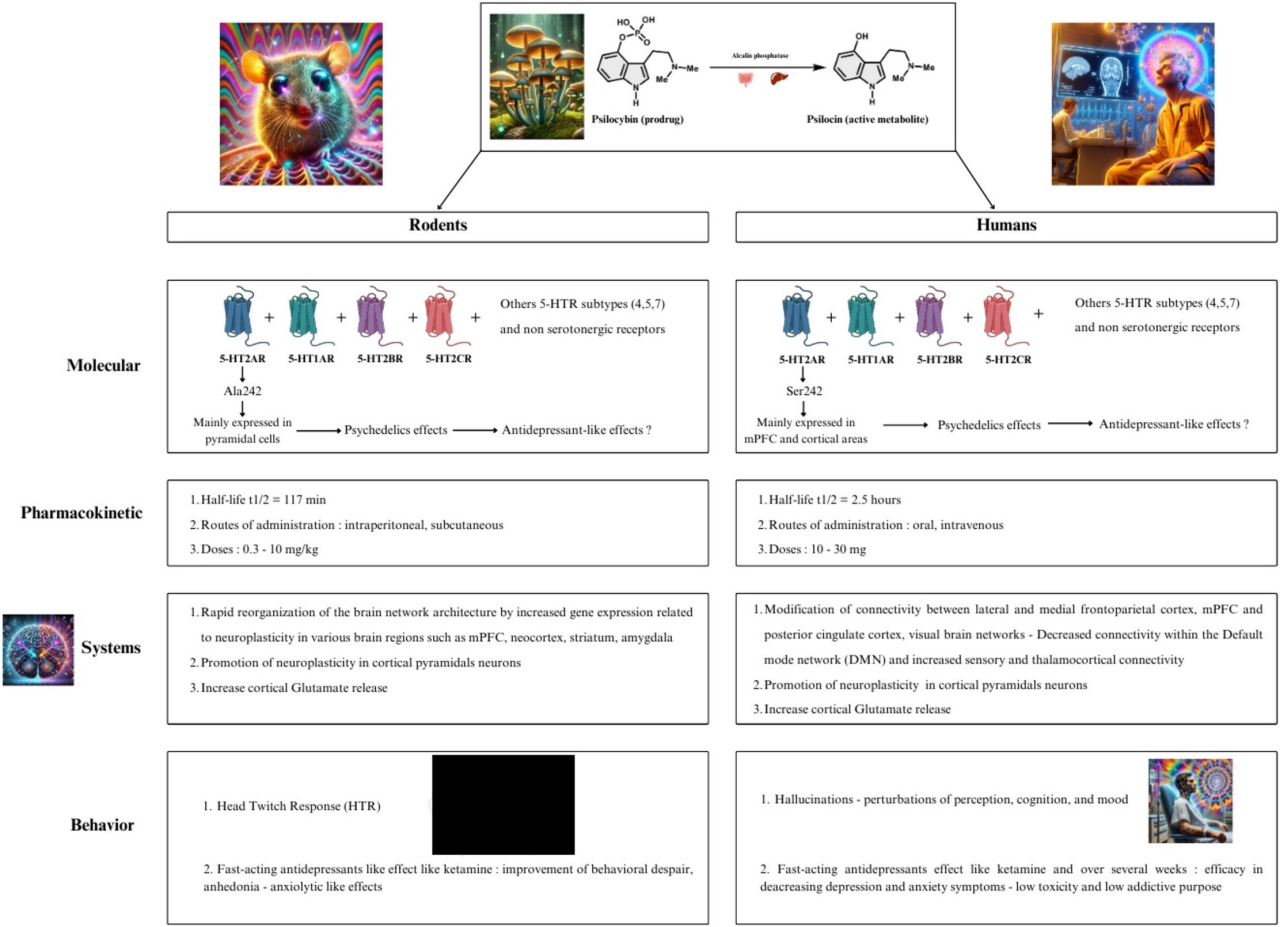
Comparison of psilocin effects on molecular, pharmacokinetic, brain neuronal systems, and at behavioral level in rodents and humans.

Figure 1 aims to draw a parallel between current knowledge from pre‐clinical and clinical literature.

The time course of ketamine's and serotonergic psychedelic's antidepressant effect is consistent with our understanding of how these drugs rewire neural circuitry and function over time. A short stimulation period (< 3 h) is sufficient for them to promote cortical neuron growth mechanisms that can last for several days or weeks [19, 156]. However, despite this different information, the preclinical studies on the mechanism of action of psychedelics (e.g., psilocybin/psilocin) are still in its infancy. Collectively, these findings suggest that many 5‐HT2AR agonists differentially activate PLC or β‐arrestin signaling pathways, stabilizing different active receptor states. When a certain signaling pathway is preferentially activated over the others, it could lead to varying qualities of efficacy for full, partial, inverse, or biased 5‐HT2AR agonists such as psychedelics [78].

Synthesis of chemical analogs that retain therapeutic properties without causing hallucinations is extremely necessary. For one, the therapeutic use of 5‐HT2AR ligands must be supervised by a health professional, usually in a hospital or other clinical setting. Due to the intense subjective effects of these drugs and a risk of a bad trip, healthcare professionals must provide support before, during, and after treatment to ensure patients that no harm comes to them during the altered state of consciousness and help them integrate their experience, usually over the course of hours. This limitation makes it unlikely that psychedelics will ever become widespread treatments for MDD and TRD.

Recent experimental findings suggest that the antidepressant and plasticity‐promoting effects of psilocybin may be dissociable from its hallucinogenic effects, and the present review sought to outline several studies that may bring us closer to understanding these potentially divergent mechanisms. Strategies for rewiring pathological neural circuitry without producing psychedelic‐like mystical responses are promising as potential treatments for patients with MDD and TRD. Whether such non‐hallucinogenic psychedelic analogs with fast‐acting antidepressant activity will be the next generation of TRD therapy is to be determined. It is currently a debate whether a profound subjective existential experience and 5‐HT2AR activation in humans are necessary for psilocybin/psilocin to display long‐lasting antidepressant effects [123]. Care should be taken in future work to correlate preclinical work that elucidates mechanisms and clinical work that seriously considers the benefits and risks of psychedelics with and without hallucinogenic effects. These future results will have important consequences for increasing some patients with TRD access to the next generation of medicines developed following serotonergic psychedelic research [111].

## Author Contributions

All authors contributed to the design of the review paper. All the authors read and approved the manuscript.

## Conflicts of Interest

The authors declare no conflicts of interest.

## Data Availability

The data that support the findings of this study are available on request from the corresponding author. The data are not publicly available due to privacy or ethical restrictions.
